# Supplementary material for: Effects of Injection Volume and Route of Administration on Dolutegravir In Situ Forming Implant Pharmacokinetics
Source: Pharmaceutics. 2022 Mar 11;14(3):615. doi: 10.3390/pharmaceutics14030615 (PMC8948873; doi:10.3390/pharmaceutics14030615)
Supplement: Supplementary file 1 [file pharmaceutics-14-00615-s001.zip › pharmaceutics-1626632-supplementary.pdf]

pharmaceutics

MDPI

Supplementary Materials

Effects of Injection Volume and Route of Administration on Dolutegravir In Situ Forming Implant Pharmacokinetics

Jordan B. Joiner, Jasmine L. King, Roopali Shrivastava, Sarah Anne Howard, Mackenzie L. Cottrell, Angela D. M. Kashuba, Paul A. Dayton and Soumya Rahima Benhabbour

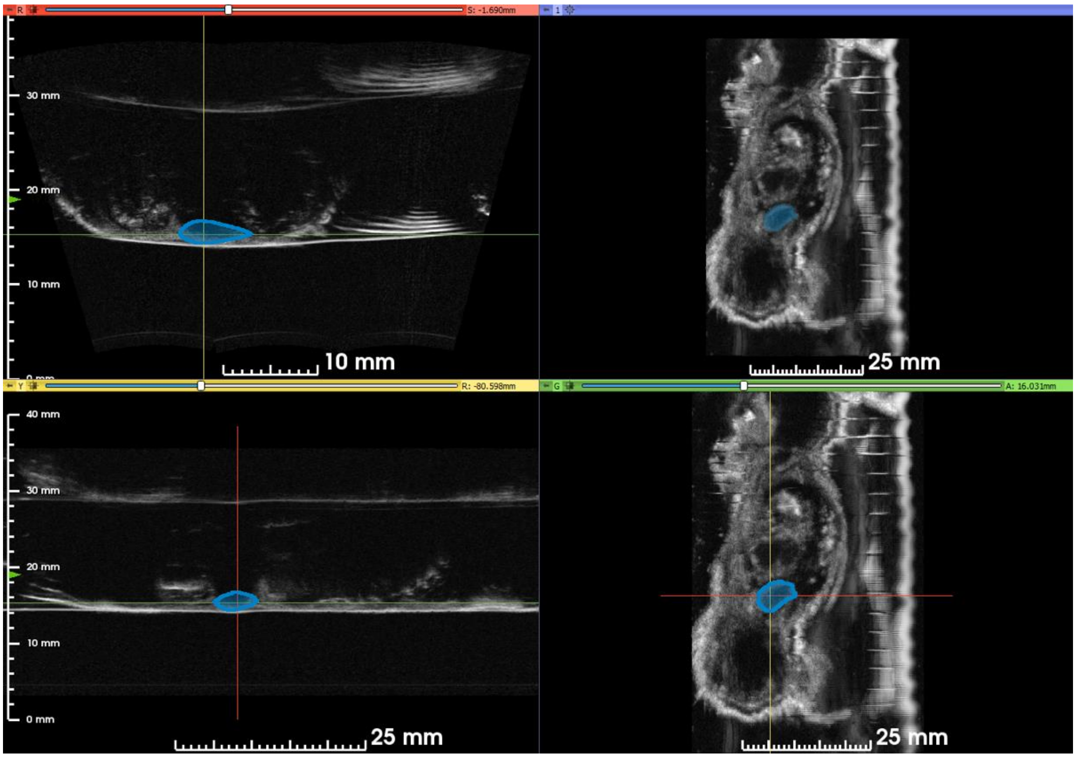

Figure S1. Representative image of the B mode ultrasound image segmentation process in SonoEQ software for a 1 × 80 µl DTG-loaded ISFI (1:2 w/w PLGA/NMP) implant injected into female NSG mouse. The red line indicates the coronal slice location, the yellow line indicates the sagittal slice location, and the green line indicates the axial slice location. (A) Interpolated ISFI segmentation in the coronal slice (B) 3-D view of the interpolated ISFI segmentation (C) Interpolated ISFI segmentation in the sagittal slice (D) Interpolated ISFI segmentation in the axial slice.

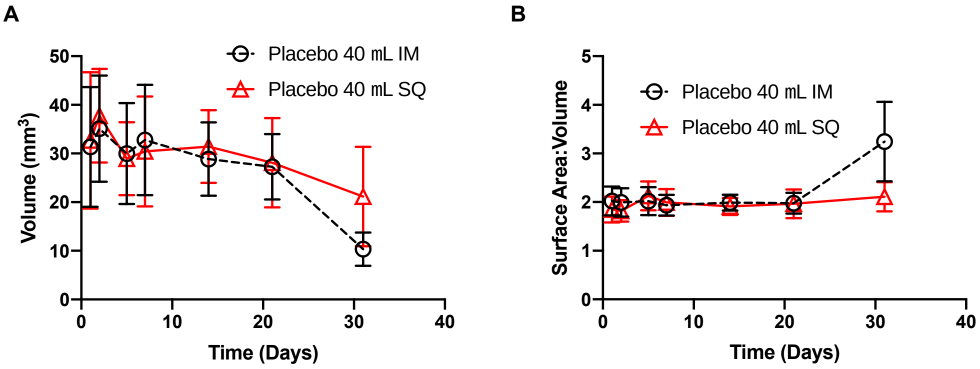

Figure S2. Comparison of 40 µl (SQ: n = 5 mice/group; n = 2 implants/mouse; IM: n = 10 mice/group; n = 1 implant/mouse) placebo ISFI (1:2 w/w PLGA/NMP) implants injected into female NSG mice.

(A) Volume of 40 µl IM and SQ placebo ISFIs over 30 days. (B) Surface area to volume ratio of 40 µl IM and SQ placebo ISFIs over 30 days.

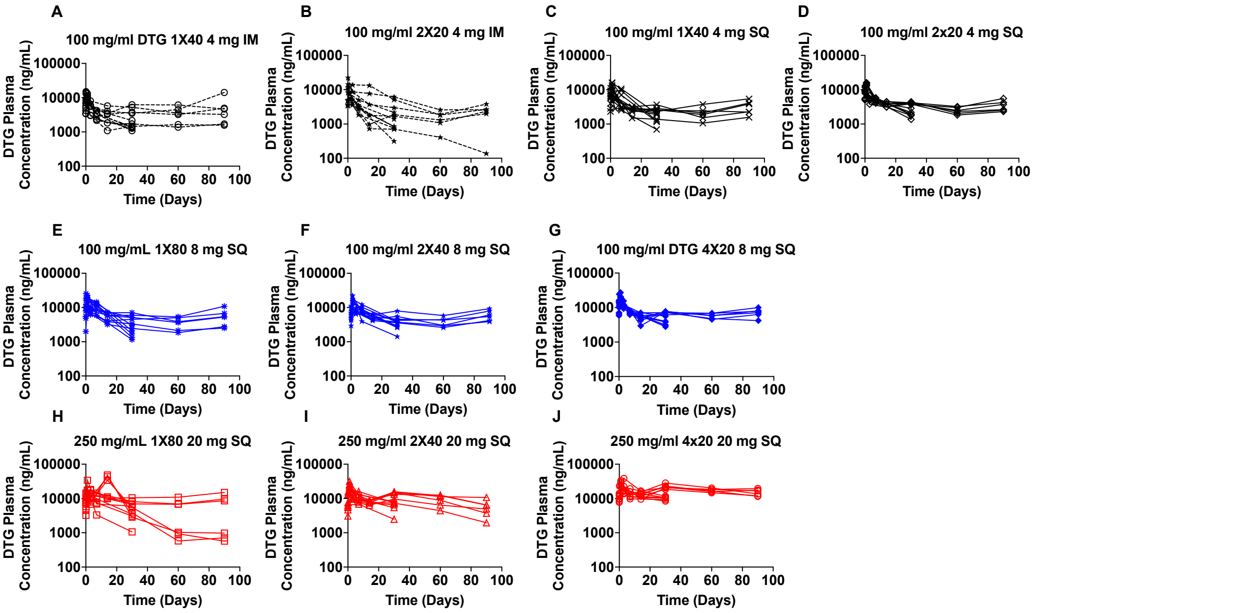

Figure S3. In vivo pharmacokinetics of DTG ISFIs (1:2 w/w PLGA/NMP) from individual female NSG mice (n = 10 mice/group). (A) 100 mg/ml 1 × 40 µl IM ISFIs (B) 100 mg/ml 2 × 20 µl IM ISFIs (C) 100 mg/ml 1 × 40 µl SQ ISFIs (D) 100 mg/ml 2 × 20 µl SQ ISFIs (E) 100 mg/ml 1 × 80 µl SQ ISFIs (F) 100 mg/ml 2 × 40 µl SQ ISFIs (G) 100 mg/ml 4 × 20 µl SQ ISFIs (H) 250 mg/ml 1 × 80 SQ ISFIs (I) 250 mg/ml 2 × 40 SQ ISFIs (J) 250 mg/ml 4 × 20 µl SQ ISFIs.

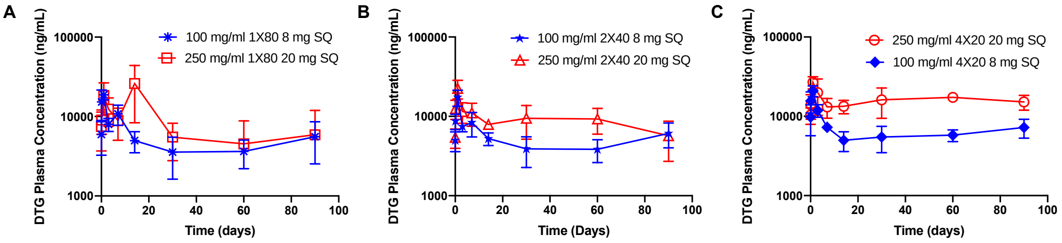

Figure S4. PK of 100 mg/ml and 250 mg/ml DTG-loaded ISFIs (1:2 w/w PLGA/NMP) injected into female NSG mice (n = 10 mice/group). Each graph compares the effect of injection volume when varying drug loading from 100 mg/ml to 250 mg/ml DTG injected SQ. (A) PK of 1 × 80 µl ISFI injections. (B) PK of 2 × 40 µl ISFI injections. (C) PK of 4 × 20 µl ISFI injections.

Table S1. Summary table of partial AUC analysis for individual rodents through day 30.

Group	Rodent_ID	AUC 0-30d
100 mg/ml 4×20 8 mg s.c.	1.00	5209420.00
	2.00	5415020.00
	3.00	5256880.00
	4.00	5012540.00
	5.00	5816460.00
	6.00	5790730.00
	7.00	6864150.00
	8.00	5935410.00
	9.00	11523840.00
	10.00	6667800.00

	11.00	7043520.00
	12.00	6529920.00
	**Median**	**5875935.00**
	**25%**	**5296415.00**
	**75%**	**6815062.50**
100 mg/ml 2×40 8 mg s.c.	13.00	5174070.00
	14.00	3667860.00
	15.00	4642820.00
	16.00	6899390.00
	17.00	5231870.00
	18.00	5699120.00
	19.00	3595800.00
	20.00	4283025.00
	21.00	3582435.00
	22.00	3996525.00
	23.00	5467905.00
	24.00	4228005.00
	**Median**	**4462922.50**
	**25%**	**3750026.25**
	**75%**	**5408896.25**
100 mg/ml 1×80 8 mg s.c.	25.00	6173120.00
	26.00	4375730.00
	27.00	7211930.00
	28.00	5564720.00
	29.00	6188170.00
	30.00	6901030.00
	31.00	4102320.00
	32.00	3292290.00
	33.00	3509235.00
	34.00	5154390.00
	35.00	5451495.00
	36.00	4872900.00
	**Median**	**5302942.50**
	**25%**	**4170672.50**
	**75%**	**6184407.50**
100 mg/ml 2×40 4 mg s.c.	37.00	3742180.00
	38.00	3226260.00
	39.00	3358140.00
	40.00	3806360.00
	41.00	3548340.00
	42.00	3757320.00
	43.00	2878920.00
	44.00	3476655.00
	45.00	3490095.00
	46.00	3422580.00
	47.00	3479160.00
	48.00	2806050.00
	**Median**	**3477907.50**
	**25%**	**3259230.00**
	**75%**	**3693720.00**
100 mg/ml 1×40 4 mg s.c.	49.00	5633570.00
	50.00	4124750.00
	51.00	4437560.00
	52.00	2828470.00

	53.00	2180526.00
	54.00	1973695.00
	55.00	2170080.00
	56.00	2353695.00
	57.00	2571795.00
	58.00	2812590.00
	59.00	2883765.00
	60.00	2472390.00
	**Median**	**2692192.50**
	**25%**	**2223818.25**
	**75%**	**3814503.75**
250 mg/ml 4×20 20 mg s.c.	61.00	9563840.00
	62.00	9504990.00
	63.00	7988520.00
	64.00	10341550.00
	65.00	12025660.00
	66.00	8961670.00
	67.00	10467150.00
	68.00	13276800.00
	69.00	11456700.00
	70.00	11591760.00
	71.00	16205850.00
	72.00	11264700.00
	**Median**	**10865925.00**
	**25%**	**9519702.50**
	**75%**	**11917185.00**
250 mg/ml 2×40 20 mg s.c.	73.00	6306400.00
	74.00	5282100.00
	75.00	9447400.00
	76.00	8700250.00
	77.00	6502470.00
	78.00	8916600.00
	79.00	6060240.00
	80.00	5550405.00
	81.00	8455530.00
	82.00	7706550.00
	83.00	7215600.00
	84.00	8287800.00
	**Median**	**7461075.00**
	**25%**	**6121780.00**
	**75%**	**8639070.00**
250 mg/ml 1×80 20 mg s.c.	85.00	2967930.00
	86.00	6874450.00
	87.00	4889110.00
	88.00	6923920.00
	89.00	8644170.00
	90.00	5900050.00
	91.00	13552530.00
	92.00	16557450.00
	93.00	17662695.00
	94.00	8575650.00
	95.00	8430780.00
	96.00	7452900.00
	**Median**	**7941840.00**

	**25%**	**6143650.00**
	**75%**	**12325440.00**
100 mg/ml 2×20 4 mg i.m.	97.00	3038395.00
	98.00	1938817.00
	99.00	1894745.00
	100.00	1339455.00
	101.00	2299090.00
	102.00	1975879.00
	103.00	2061270.00
	104.00	1649661.00
	105.00	4026165.00
	106.00	8029950.00
	107.00	5669145.00
	108.00	1393065.00
	**Median**	**2018574.50**
	**25%**	**1710932.00**
	**75%**	**3779222.50**
100 mg/ml 1×40 4 mg i.m.	109.00	2433510.00
	110.00	3094930.00
	111.00	2144470.00
	112.00	2249010.00
	113.00	2301000.00
	114.00	2245095.00
	115.00	1714650.00
	116.00	3555510.00
	117.00	3365265.00
	118.00	3298815.00
	119.00	1456275.00
	120.00	4742055.00
	**Median**	**2367255.00**
	**25%**	**2169626.25**
	**75%**	**3348652.50**

Table S2. Pooled 90-day NCA analysis for each treatment group.

Group	Dose (mg)	C_{max} (ng/ml)	SE_{Cmax} ^{a} (ng/ml)	T_{max} (hr)	AUC_{all} (hr*ng/ml)	SE_{AUCall} ^{a} (hr*ng/ml)
100 mg/ml 4×20 s.c.	8	20916.67	1329.77	24.00	15501924.17	1015938.51
100 mg/ml 2×40 s.c.	8	17383.33	1618.11	24.00	10912253.33	957211.45
100 mg/ml 1×80 s.c.	8	18766.67	1022.96	24.00	10797044.17	1129956.20
250 mg/ml 4×20 s.c.	20	26883.33	1925.52	24.00	34759638.33	1650513.36
250 mg/ml 2×40 s.c.	20	22500.00	2423.22	24.00	19355618.75	1902268.03
250 mg/ml 1×80 s.c.	20	26080.00	7250.18	336.00	18438518.33	1198904.52
100 mg/ml 2×20 s.c.	4	14600.00	508.59	24.00	7434050.00	404444.56
100 mg/ml 1×40 s.c.	4	11888.33	1718.43	24.00	6214291.00	568354.27

100 mg/ml 2×20 i.m.	4	12638.33	2164.93	3.00	5721920.83	1194308.24
100 mg/ml 1×40 i.m.	4	11366.67	1268.22	3.00	7839729.17	1370045.07

[a] SE denotes standard error estimate for each PK parameter

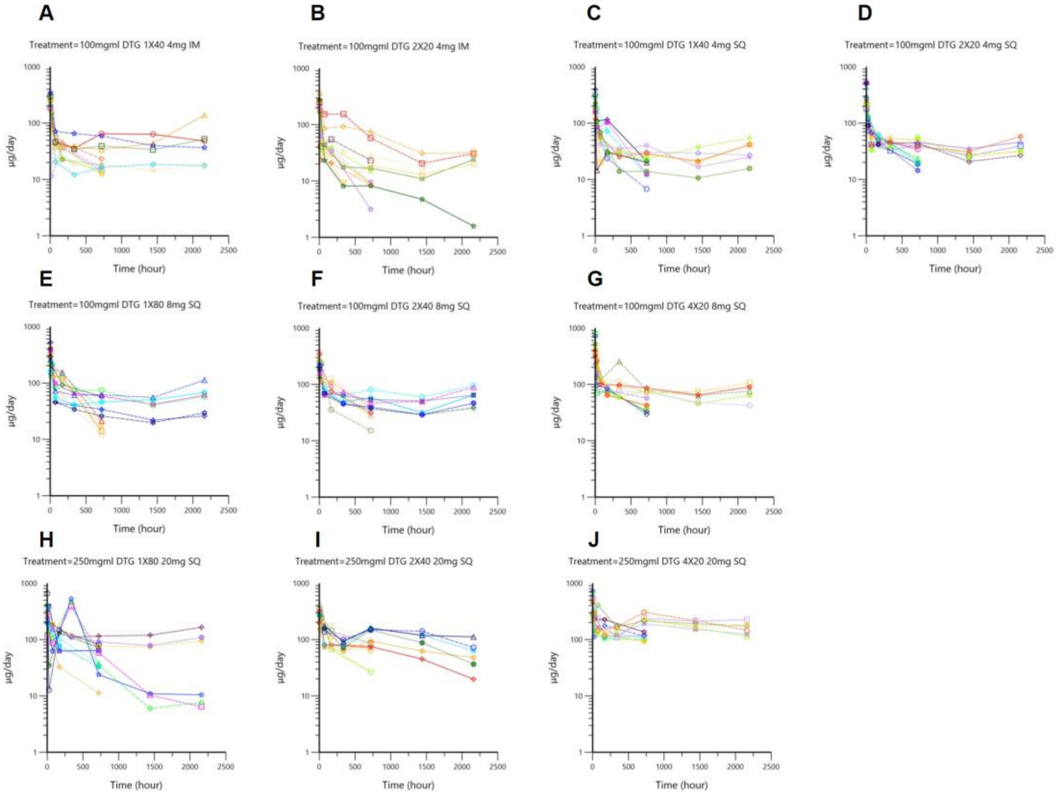

Figure S5. Release rates of DTG ISFIs (1:2 w/w PLGA/NMP) from individual female NSG mice (n = 10 mice/group) as modeled by deconvolution analysis. (A) 100 mg/ml 1 × 40 µl IM ISFIs (B) 100 mg/ml 2 × 20 µl IM ISFIs (C) 100 mg/ml 1 × 40 µl SQ ISFIs (D) 100 mg/ml 2 × 20 µl SQ ISFIs (E) 100 mg/ml 1 × 80 µl SQ ISFIs (F) 100 mg/ml 2 × 40 µl SQ ISFIs (G) 100 mg/ml 4 × 20 µl SQ ISFIs (H) 250 mg/ml 1 × 80 SQ ISFIs (I) 250 mg/ml 2 × 40 SQ ISFIs (J) 250 mg/ml 4 × 20 µl SQ ISFIs.

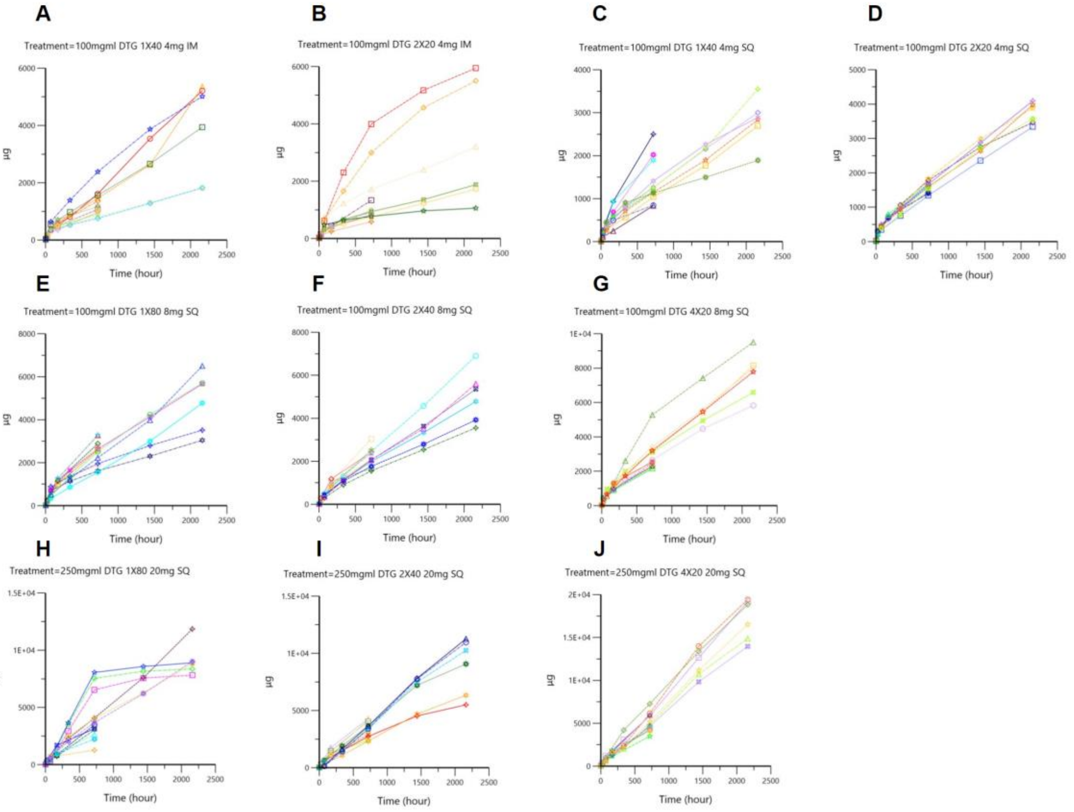

Figure S6. Cumulative release of DTG from ISFIs (1:2 w/w PLGA/NMP) from individual female NSG mice (n = 10 mice/group) as modeled by deconvolution analysis. (A) 100 mg/ml 1 × 40 µl IM ISFIs (B) 100 mg/ml 2 × 20 µl IM ISFIs (C) 100 mg/ml 1 × 40 µl SQ ISFIs (D) 100 mg/ml 2 × 20 µl SQ ISFIs (E) 100 mg/ml 1 × 80 µl SQ ISFIs (F) 100 mg/ml 2 × 40 µl SQ ISFIs (G) 100 mg/ml 4 × 20 µl SQ ISFIs (H) 250 mg/ml 1 × 80 SQ ISFIs (I) 250 mg/ml 2 × 40 SQ ISFIs (J) 250 mg/ml 4 × 20 µl SQ ISFIs.

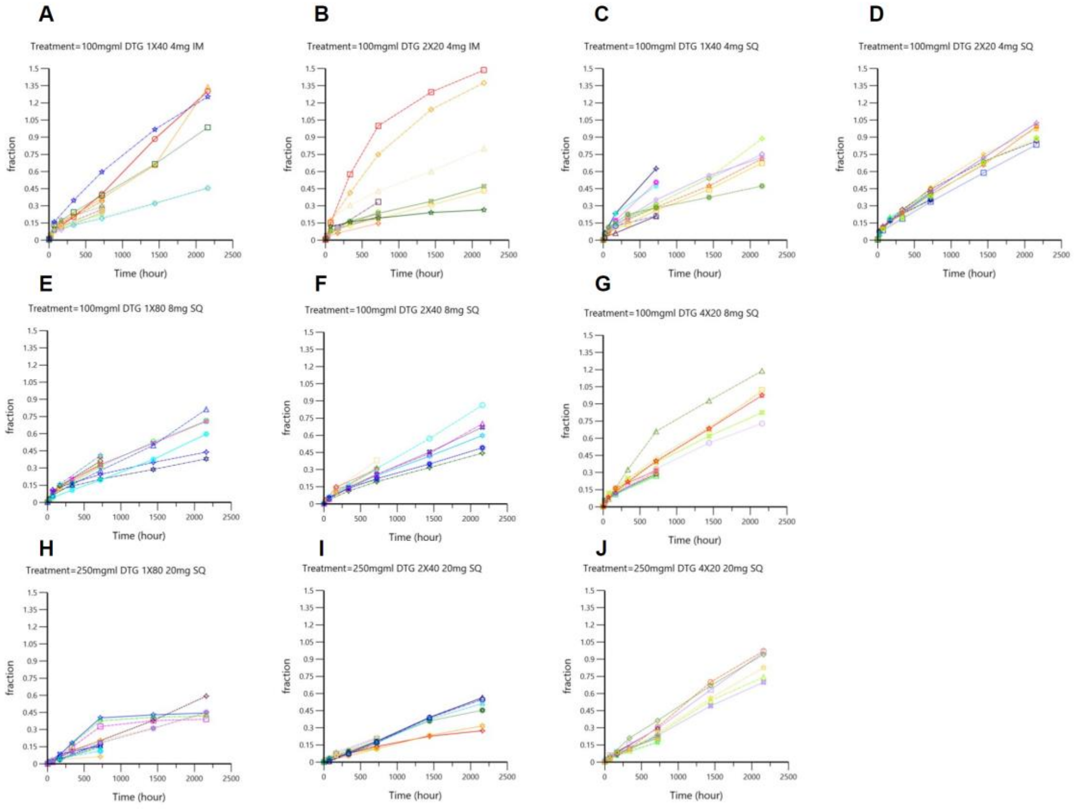

Figure S7. Fraction of DTG released from ISFIs (1:2 w/w PLGA/NMP) from individual female NSG mice (n = 10 mice/group) as modeled by deconvolution analysis. (A) 100 mg/ml 1 × 40 µl IM ISFIs (B) 100 mg/ml 2 × 20 µl IM ISFIs (C) 100 mg/ml 1 × 40 µl SQ ISFIs (D) 100 mg/ml 2 × 20 µl SQ ISFIs (E) 100 mg/ml 1 × 80 µl SQ ISFIs (F) 100 mg/ml 2 × 40 µl SQ ISFIs (G) 100 mg/ml 4 × 20 µl SQ ISFIs (H) 250 mg/ml 1 × 80 SQ ISFIs (I) 250 mg/ml 2 × 40 SQ ISFIs (J) 250 mg/ml 4 × 20 µl SQ ISFIs.

Table S3. Median estimated deconvolution parameters of 4 × 20, 2 × 40, and 1 × 80 µl DTG-loaded ISFIs (1:2 w/w PLGA:NMP).

		Variable		
		**Fraction Input**	**µg**	**µg/day**
**Treatment**	**Time (hour)**	**Median**		
100 mg/ml DTG 1 × 40 4mg IM	1.00	0.00	11.69	252.86
	3.00	0.01	30.73	230.80
	24.00	0.05	185.30	77.69
	72.00	0.10	406.17	41.67
	168.00	0.12	489.22	36.72
	336.00	0.21	845.15	35.06
	720.00	0.28	1127.56	18.05
	1440.00	0.66	2641.25	36.79
	2160.00	1.12	4479.55	42.36
	**Min**	**0.00**	**11.69**	**18.05**
	**Max**	**1.12**	**4479.55**	**252.86**
100 mg/ml DTG 1 × 40 4mg SQ	1.00	0.00	11.55	245.03
	3.00	0.01	22.64	170.96
	24.00	0.05	182.99	91.66
	72.00	0.09	345.66	55.00
	168.00	0.16	636.40	51.53
	336.00	0.20	805.36	28.98

	720.00	0.30	1213.66	21.30
	1440.00	0.51	2028.16	20.72
	2160.00	0.72	2864.04	34.28
	**Min**	**0.00**	**11.55**	**20.72**
	**Max**	**0.72**	**2864.04**	**245.03**
100 mg/ml DTG 1 × 80 8mg SQ	1.00	0.00	13.78	310.60
	3.00	0.01	42.51	321.89
	24.00	0.03	250.17	178.37
	72.00	0.08	606.46	63.46
	168.00	0.14	1107.85	126.46
	336.00	0.16	1286.80	50.86
	720.00	0.32	2552.86	33.59
	1440.00	0.44	3492.47	41.05
	2160.00	0.65	5221.91	58.92
	**Min**	**0.00**	**13.78**	**33.59**
	**Max**	**0.65**	**5221.91**	**321.89**
100 mg/ml DTG 2 × 20 4mg IM	1.00	0.00	10.66	225.89
	3.00	0.01	34.77	264.38
	24.00	0.04	143.47	44.60
	72.00	0.14	541.04	49.02
	168.00	0.10	397.55	33.79
	336.00	0.24	942.28	25.88
	720.00	0.23	931.41	15.99
	1440.00	0.47	1879.25	15.32
	2160.00	0.64	2542.03	27.39
	**Min**	**0.00**	**10.66**	**15.32**
	**Max**	**0.64**	**2542.03**	**264.38**
100 mg/ml DTG 2 × 20 4mg SQ	1.00	0.00	16.65	358.00
	3.00	0.01	29.20	220.66
	24.00	0.06	238.09	104.59
	72.00	0.11	425.83	40.94
	168.00	0.18	704.03	52.14
	336.00	0.24	941.50	43.86
	720.00	0.40	1605.80	36.25
	1440.00	0.68	2739.23	26.87
	2160.00	0.93	3724.21	35.45
	**Min**	**0.00**	**16.65**	**26.87**
	**Max**	**0.93**	**3724.21**	**358.00**
100 mg/ml DTG 2 × 40 8mg SQ	1.00	0.00	11.95	269.08
	3.00	0.00	26.56	202.16
	24.00	0.03	228.17	150.24
	72.00	0.05	418.20	70.28
	168.00	0.13	1003.98	87.63
	336.00	0.14	1116.60	56.09
	720.00	0.26	2074.43	39.25
	1440.00	0.43	3428.56	40.71
	2160.00	0.63	5070.64	64.72
	**Min**	**0.00**	**11.95**	**39.25**
	**Max**	**0.63**	**5070.64**	**269.08**
100 mg/ml DTG 4 × 20 8mg SQ	1.00	0.00	20.79	460.85
	3.00	0.01	42.47	322.96
	24.00	0.04	336.09	139.82
	72.00	0.08	644.54	116.84

	168.00	0.12	972.13	78.12
	336.00	0.23	1876.75	94.63
	720.00	0.32	2599.09	62.61
	1440.00	0.68	5444.06	63.85
	2160.00	0.99	7908.07	81.44
	**Min**	**0.00**	**20.79**	**62.61**
	**Max**	**0.99**	**7908.07**	**460.85**
250 mg/ml DTG 1 × 80 20mg SQ	1.00	0.00	18.63	402.74
	3.00	0.00	28.53	219.79
	24.00	0.01	275.94	133.08
	72.00	0.03	503.56	128.20
	168.00	0.05	900.77	91.11
	336.00	0.14	2700.46	255.39
	720.00	0.18	3572.21	61.21
	1440.00	0.38	7580.87	42.64
	2160.00	0.44	8851.90	52.45
	**Min**	**0.00**	**18.63**	**42.64**
	**Max**	**0.44**	**8851.90**	**402.74**
250 mg/ml DTG 2 × 40 20mg SQ	1.00	0.00	14.26	325.55
	3.00	0.00	35.49	271.60
	24.00	0.01	269.01	239.59
	72.00	0.02	443.21	108.55
	168.00	0.06	1232.45	120.55
	336.00	0.07	1464.62	79.93
	720.00	0.17	3377.31	86.01
	1440.00	0.37	7352.62	103.26
	2160.00	0.48	9663.33	55.73
	**Min**	**0.00**	**14.26**	**55.73**
	**Max**	**0.48**	**9663.33**	**325.55**
250 mg/ml DTG 4 × 20 20mg SQ	1.00	0.00	23.85	542.04
	3.00	0.00	35.04	277.18
	24.00	0.02	413.79	249.53
	72.00	0.03	641.94	153.63
	168.00	0.08	1608.00	119.53
	336.00	0.11	2271.83	138.20
	720.00	0.24	4774.83	164.35
	1440.00	0.60	11912.41	180.02
	2160.00	0.88	17684.01	159.70
	**Min**	**0.00**	**23.85**	**119.53**
	**Max**	**0.88**	**17684.01**	**542.04**

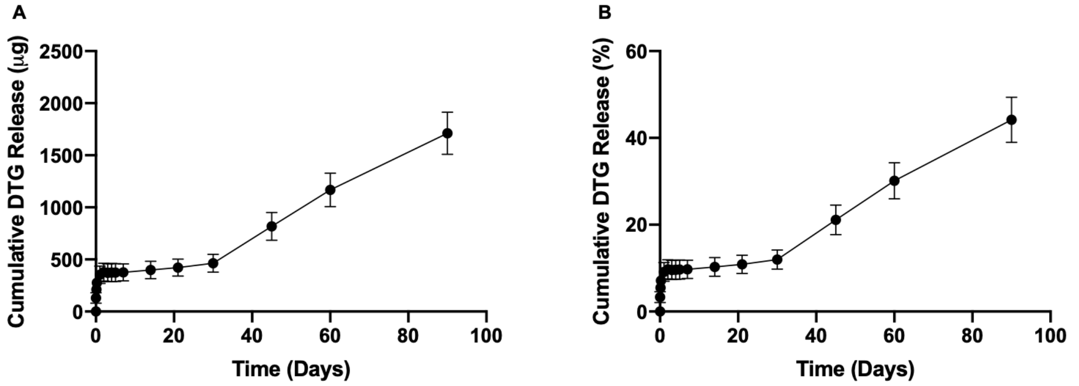

C

Formulation	Volume (µl)	Analytical Drug Loading (µg/mg)	Release/day at zero order (Days 30-90)
100 mg/ml DTG in 1:2 PLGA:DMSO	40	100.0 ± 2.53	20.7 µg/day (R^2 = 0.99)

Figure S8. In vitro drug release of 40 µl 100 mg/ml DTG ISFIs (n=4) over 90 days in a 1:2 PLGA:DMSO polymer-solvent system shown as cumulative DTG release in µg (a) and cumulative DTG release as a percentage of initial drug loaded (b).

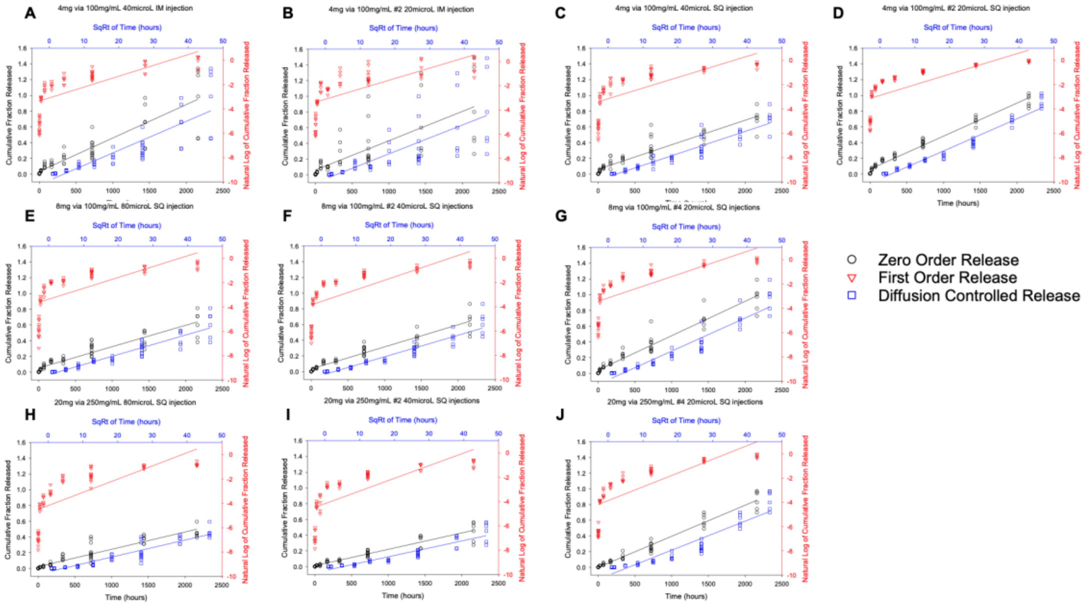

Figure S9. Fraction of DTG released from ISFIs (1:2 w/w PLGA/NMP) from individual female NSG mice (n = 10 mice/group) fit to zero order, first order, and diffusion-controlled release models. (A) 100 mg/ml 1 × 40 µl IM ISFIs (B) 100 mg/ml 2 × 20 µl IM ISFIs (C) 100 mg/ml 1 × 40 µl SQ ISFIs (D) 100 mg/ml 2 × 20 µl SQ ISFIs (E) 100 mg/ml 1 × 80 µl SQ ISFIs (F) 100 mg/ml 2 × 40 µl SQ ISFIs (G) 100 mg/ml 4 × 20 µl SQ ISFIs (H) 250 mg/ml 1 × 80 SQ ISFIs (I) 250 mg/ml 2 × 40 SQ ISFIs (J) 250 mg/ml 4 × 20 µl SQ ISFIs.

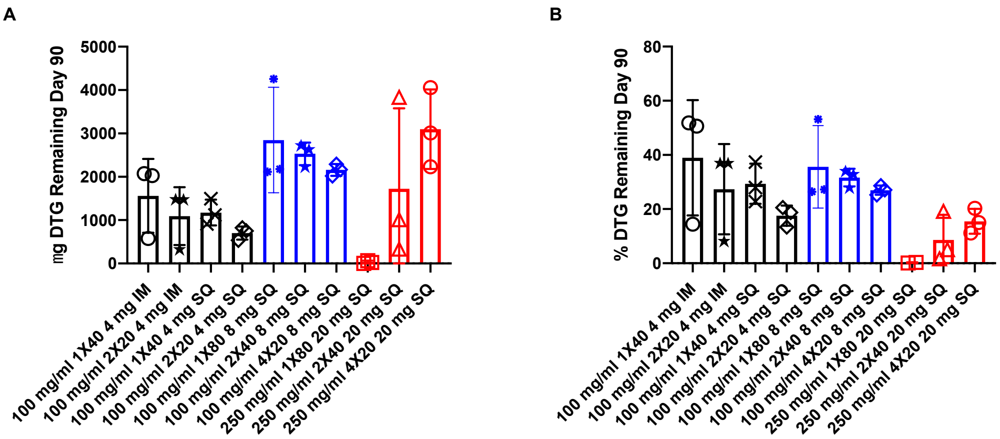

Figure S10. Residual DTG concentrations in implants injected into female NSG mice (n=3/group) and retrieved at day 90 post-implantation determined by fully dissolving implants in ACN overnight and quantifying DTG concentration by HPLC. (A) µl of DTG remaining in ISFIs at day 90 (B) Percent DTG remaining in ISFI at day 90 based on total administered dose.
